# Extracellular Vesicles: New Tools for Early Diagnosis of Breast and Genitourinary Cancers

**DOI:** 10.3390/ijms22168430

**Published:** 2021-08-05

**Authors:** Anna Testa, Emilio Venturelli, Maria Felice Brizzi

**Affiliations:** Department of Medical Sciences, University of Turin, 10126 Turin, Italy; anna.testa@unito.it (A.T.); emilio.venturelli@edu.unito.it (E.V.)

**Keywords:** oncology, extracellular vesicles, liquid biopsy

## Abstract

Breast cancers and cancers of the genitourinary tract are the most common malignancies among men and women and are still characterized by high mortality rates. In order to improve the outcomes, early diagnosis is crucial, ideally by applying non-invasive and specific biomarkers. A key role in this field is played by extracellular vesicles (EVs), lipid bilayer-delimited structures shed from the surface of almost all cell types, including cancer cells. Subcellular structures contained in EVs such as nucleic acids, proteins, and lipids can be isolated and exploited as biomarkers, since they directly stem from parental cells. Furthermore, it is becoming even more evident that different body fluids can also serve as sources of EVs for diagnostic purposes. In this review, EV isolation and characterization methods are described. Moreover, the potential contribution of EV cargo for diagnostic discovery purposes is described for each tumor.

## 1. Introduction

Cancer is a major public health problem representing the second cause of death worldwide after cardiovascular diseases. In total, 19.3 million new cancer cases (18.1 million excluding non-melanoma skin cancer) and almost 10.0 million cancer deaths (9.9 million excluding non-melanoma skin cancer) were estimated worldwide in 2020 [1]. Breast cancer is described as the most commonly diagnosed cancer in women and the leading cause of cancer-related death in the female population [1,2]. In addition, gynecological malignancies, including ovarian cancer, endometrial cancer, and genitourinary neoplasms, such as prostate, bladder, and kidney neoplasms, greatly contribute to the cancer burden globally [3,4,5,6,7]. In addition, the COVID-19 pandemic in 2020 has further indirectly influenced the existing cancer-associated medical and social issues [8,9]. The consequences of the delays in diagnosis and lack of patient care associated with the pandemic may change the population wellness in the upcoming years. This challenging period further highlights the need for worth cancer diagnostics able to promptly and easily detect the disease in a large population, particularly in the highly prevalent breast and prostate cancers.

Using liquid biopsy, which consists of the analysis of tumor cells and tumor derivatives in biological fluids, it may be challenging to hastily process a large number of clinical samples and to speed up early cancer diagnosis [10]; therefore, the discovery of novel diagnostic tools based on liquid biopsy derivatives may revolutionize the clinical practice. Indeed, extracellular vesicles (EVs) are the most promising candidates.

EVs are bilayer lipid-membrane-limited structures, which are derived from the cell membrane or from the cytoplasmic materials and released in the microenvironment by almost all cell types. EVs convey a huge variety of cellular-derived molecules, such as lipids, proteins, DNA, messenger RNAs (mRNAs), microRNAs (miRNA), small non-coding RNAs (sncRNAs), and long non-coding RNAs (lncRNAs) [11].

Based on the Minimal Information for Studies of Extracellular Vesicles (MISEV) guidelines of the International Society of Extracellular Vesicles (ISEV), EVs can be classified on the basis of their size (for example small EVs or medium-large EVs), biochemical features, density, or cell of origin [12]. EVs participate in both physiological and pathological human diseases, including cancers. Specifically, in cancer, EVs play an essential role in different oncogenic processes in the tumor microenvironment (TME) and at distant sites [13,14].

Unbiased and quantifiable features of specific biological processes are required for biomarker discovery. Moreover, biomarkers should provide insight into the mechanism of the disease, should be easy to measure, inexpensive, differentially expressed in physiological and pathological settings, and detected by non-invasive procedures [15]. Since EVs can be recovered in different biological fluids, including serum, plasma urine, ascites, pleural, and pericardial effusions, their contents reflect the microenvironment of the cell of origin and express specific markers of their cell of origin. EVs hold great potential as cancer biomarkers and for use in diagnostics. In this review, current evidence and future EV-related perspectives for the diagnosis of gynecological and genitourinary cancers are discussed.

## 2. Isolation and Characterization of EVs for the Diagnosis of Gynecological and Genitourinary Malignancies

EVs that are regularly secreted by normal cells into the extracellular space can be recovered in many if not all biological fluids [16,17]. More importantly, specific cancer-cell-derived EVs are released into the extracellular space and can be found in plasma, serum, and urine of tumor-bearing patients. The ability to properly isolate EVs without altering their structural and biological features represents an important step to implement EV-based cancer diagnostic strategies. After isolation, many molecular biology techniques can be applied to characterize patient-derived EVs. The characterization process should ideally be able to identify specific features of cancer-derived EV, such as miRNAs, proteins, or DNA, to precisely distinguish cancer from healthy individuals. In this paragraph, we discuss the sources and methods used to isolate and characterize EVs relevant for the diagnosis of breast, gynecological, and genitourinary cancers.

Ultracentrifugation (UC), density gradient centrifugation (DC), precipitation, immunoaffinity capture, size exclusion chromatography (SEC), and microfluidic-based isolation are the most relevant EV isolation procedures for patients with suspected cancer [17,18]. UC has historically been regarded as the gold standard for EV isolation in basic and translational research. This consists of low-speed centrifugation (300× *g* for 10 min) aiming to eliminate dead cells and apoptotic bodies followed by high speed centrifugation (100,000× *g* for 2 h) [19]. Even if UC is a widely used method in EV research, several issues limit its clinical application. Indeed, UC-based isolation does not guarantee sufficient EV purity, particularly for EV-associated proteins. DC is similar to UC, although increasing concentrations of sucrose or iodixonal are added on the top of the gradient before the centrifugation process [20]. The EV purity using DC is higher than using UC, since it better eliminates contaminating proteins [19]; however, both UC and DC are time-consuming and time-demanding, making their clinical application difficult. Precipitation (e.g., including commercially available kits such as ExoQuick) involves the use of polyethylene glycol (PEG) to pellet EVs at lower centrifugation speeds [21]. It is less time-consuming than UC and DC, although the risk of protein contamination is higher and further purification procedures should be adopted to obtain a good standard of purity. The study by Zhang et al. [20] compared UC and DG to isolate human cancer cell line (TCA8813) EVs. DG-derived EVs were found to be uniform in size and more enriched in EV-specific proteins, indicating a higher isolation efficiency than UC; however, routine clinical application of DG is limited by its cost, the need for technical skills, and the time needed for the isolation process. Tauro and colleagues [19] compared UC, DG, and the immunoaffinity capture method for the isolation of human cancer cell line (LIM1863)-derived EVs. EVs obtained from culture media using these different methods have been analysed using Western blot, transmission electron microscopy (TEM), and liquid chromatography tandem mass spectrometry (GeLC-MS/MS). All methods were able to isolate EVs ranging from 40 to 100 nm; however, the immunoaffinity capture technique was considered superior, since EV markers and EV-associated proteins were enriched at least two-fold compared to UC and DG. An additional advantage of the immunoaffinity-based isolation method is in its ability to selectively capture EVs based on their source and specific surface markers (e.g., from cancer cells). SEC is able to separate EVs based on their size and hydrodynamic diameter [16,22]. SEC uses a column for chromatographic separation and has been shown to isolate EVs from a large variety of body fluids, such as plasma, serum, or urine (making it a potential non-invasive approach for renal and prostate cancer screening and diagnosis). As proposed by Böing et al. [23], SEC can be used for rapid and simple EV isolation and purification. SEC isolation can avoid the enrichment of additional proteins, which is a relevant step for their clinical application; however, the potential limitations of this technique include the enrichment of non-EV nanoparticles (e.g., particles lacking tetraspanins) displaying similar hydrodynamic diameters. In fact, the ability of SEC to distinguish EVs from lipoproteins with satisfactory accuracy is still debated [24].

The transfer of EVs in clinical settings has been also proposed. It has been demonstrated that EVs can be obtained from different biological fluids, such as serum, plasma, or urine. Saenz-Cuesta [25] and colleagues compared ExoQuick isolation, UC, and DC, and proposed the DC protocol for clinical practice. Likewise, Gaspar et al. [26] proposed a fast and simple protocol for SEC-based EV isolation from plasma. In addition to serum- and plasma-derived EVs, urine-derived EVs also hold diagnostic potential, especially for genitourinary and breast cancers. As demonstrated by Cheng et al. [27], 20 mL of urine is sufficient to extract and analyze EV miRNAs. More importantly, the miRNA content in urine is higher in EVs than in the cell pellet or cell free urine. In renal, bladder, and prostate cancers, urine has been proposed for diagnostic purposes [28,29,30,31,32]. Interestingly, urine can likely be used as a diagnostic tool even in non-genitourinary cancers. As an example, Hirschfeld et al. [33] investigated the role of urine EV miRNAs in the diagnosis of breast cancer, obtaining high diagnostic accuracy. This evidence, together with the risk-free, non-invasive, practitioner-friendly, and cost-efficient procedures associated with urine collection, make urine a promising diagnostic source for EV-based diagnosis.

In addition to these classic EV isolation and quantification methods, emerging techniques are attracting interest in EV research. Among these, immunoaffinity capture uses specific antibodies to detect and isolate EVs based on their surface markers [34]. The antibody–antigen recognition is of particular interest, as it can allow the direct isolation of specific EVs (e.g., cancer-derived EVs), distinguishing them from those of non-cancer origin [35]. Variants of this isolation technique, particularly microfluidic-based immunoaffinity capture, have been used to obtain EVs from patient plasma of sufficient purity for diagnostic purposes in breast cancer [36]. A similar isolation technique based on antibody-mediated specific EV antigen recognition, called proximity ligation assay, has been used to isolate prostate-cancer-derived EVs [37]. In recent years, microfluidic devices have been proposed as valid instruments for EV isolation. The microfluidic isolation technique has attracted particular interest in this field since it is able to isolate EVs based on (a) size, (b) density, and (c) surface antigens (microfluidic immunoaffinity capture) [38,39]; however, no definitive data on their potential clinical application are so far available. EV characterization and analysis are also crucial for their diagnostic application, since the aim is to detect specific EV-associated cancer biomarkers. An ideal biomarker should be cancer-type-specific and its diagnostic value should be validated by large-scale diagnostic studies; therefore, depending on the specific biomarker, different characterization methods can be applied. An enzyme-linked immune assay (ELISA) can be used for the detection and quantification of EV-related proteins, flow cytometry (FC) can be used for surface antigen identification, while OMICS-based techniques can be used for nucleic acid detection in EV cargo. Several examples in breast, genitourinary, and ovarian cancer are discussed in this review, while a more detailed description of EV analysis methods is available in [40,41,42].

## 3. Breast Cancer

Breast cancer (BC) is the most common type of tumor among women and the main cause of cancer-related mortality around the world [2,43]. In recent years, EVs have been progressively recognized as fundamental players in breast cancer development and metastasis [44]; thus, there is a constant demand for the development of new diagnostic tools, including EVs. ELISA and qRT-PCR are the most common methodologies used to detect specific EV biomarkers based on their cargo. Proteins and RNAs, including miRNAs and long-noncoding RNAs, have mostly been investigated. For the first time, Cui et al. [45] identified the lactate dehydrogenase C (LDHC) in serum and EVs as a BC biomarker by quantifying its mRNA expression in serum, serum-derived EVs, and the protein in BC tissues. Hannafon et al. [46] demonstrated that miR-21 and miR-1246 are specifically detected in human breast cancer EVs, in plasma from both breast cancer patient-derived xenograft (PDX) mice and breast cancer patients. Li et al. [47] focused on the chromosome-X-located miR-106a–363 cluster, looking into plasma and serum EV miRNAs (miR-106a-3p, miR-106a-5p, miR-20b-5p, and miR-92a-2-5p in plasma and miR-106a-5p, miR-19b-3p, miR-20b-5p, and miR-92a-3p in serum). They demonstrated that all miRNAs except for plasma-derived miR-20b-5p were significantly upregulated in BC patients. Recently, a panel of four variable urinary miRNAs (miR-424, miR-423, miR-660, and let7-i) was identified as a diagnostic tool, displaying 98.6% sensitivity and 100% specificity in BC patients [33]. Ozawa et al. [48] have suggested a different miR panel including 3 miRNAs as potential BC biomarkers. The panel, which includes miR-142-5p, miR-320a, and miR-4433b-5p, showed an AUC corresponding to 0.8387, a sensitivity of 93.33%, and a specificity of 68.75%. Eichelser et al. [49] demonstrated that exosomal miR-101, miR-372, and miR-373 enriched in serum EVs of patients with BC are higher than in healthy controls. Moreover, miR-373 was suggested as a triple-negative breast cancer diagnostic biomarker, since it was higher in this subgroup of patients than in patients with luminal cancers or healthy controls. In a different study, Moon et al. [50] first demonstrated the enrichment of Del-1 in plasma EVs as a diagnostic marker for breast cancer in a test cohort and an independent validation cohort using two different ELISA assays. Moon et al. [51] also identified fibronectin (FN) as a valid biomarker carried by breast-cancer-derived EVs. More importantly, the authors showed that the diagnostic accuracy of FN in EVs is higher than in plasma. Kibria et al. [52] proved that a different CD47 expression in circulating EVs correlated with the BC status, even though the molecular mechanism is not yet clear.

Fang et al. [36] used a microfluidic chip for immunocapture and quantification of circulating EVs. This approach allowed the detection of significantly increased EV epithelial cell adhesion molecule (EpCAM) contents in plasma samples of BC patients. This observation was in contrast with the data from Rupp and colleagues [53], who failed to detect EpCAM in serum EVs isolated from BC patients. Since the serum proteolytic activity may explain this discrepancy, the immunocapture methodology has been proposed as a more sensitive technique. Furthermore, Fang et al. [36] demonstrated that the EV HER2 content correlates with the tumor tissue content by using a microfluidic chip. Nanou et al. [54] recently reported a similar prognostic power for EpCAM+ cytokeratin (CK) circulating tumor cells (CTCs) and tumor-derived EVs (tdEVs), as well as EpCAM+ CK+ CTCs and tdEVs in BC, by including anti-HER2 in the CellSearch assay. They showed that tdEVs better reflect the HER2 phenotype of primary tumors than CTCs. Interestingly, they also found that the presence of heterogeneous CTC and tdEVs (2 or 3 different immunophenotypes) was associated with poor survival compared to patients with uniform CTCs and tdEVs (1 immunophenotype). Domenyuk et al. [55] developed the adaptive dynamic artificial poly-ligand targeting (ADAPT) approach as a highly specific profiling tool and demonstrated that the binding profile of diverse ssODN libraries, enriched by “plasma-SELEX”, can be used to identify BC, as well as the tumor biological behavior. More recently, Moura et al. [56] tested a magneto-actuated immunoassay to detect EVs in undiluted human serum and were able to differentiate purified EVs from healthy donors and BC patients by using specific epithelial biomarkers. This tool has been proposed as an alternative and more feasible clinical approach compared to the standard flow cytometry technique. We are confident that all of these technologies have their own limitations; however, the combination of different methodologies along with clinical information derived from already established biological tests and imaging would be a future challenge for more accurate diagnosis, particularly in patients with tumor recurrence.

Kwizera et al. [57] developed a Raman-based assay for the molecular profiling of EVs and the detection of HER2 and EpCAM-EV contents, with high sensitivity and specificity. Although Raman-based technology still require standardization, this tool has been proposed for biomarker discovery in HER2-positive BC patients, being able to differentiate either different subtypes of cancer cells or cancer cells from normal cells. EV-associated biomarkers investigated for breast cancer diagnosis are reported in Table 1.

## 4. Uterine and Cervical Cancer

### 4.1. Uterine Cancer

Endometrial cancer (EC) is the most prevalent gynecological cancer in developed countries [4]. In particular, uterine mesenchymal tumors (UMT) are a heterogeneous group, comprising both benign and malignant variants and affecting over 50% of Caucasian women and up to 80% of African women. UMT are also one of the foremost causes of hysterectomy [58,59]. Srivastava et al. [60] have suggested the potential application of the EV-hsa-miR content as a biomarker for endometrial cancer diagnosis.

Dvorská et al. [61] concluded that currently there are no reliable liquid biopsy (LB) biomarkers able to distinguish specific UMT subtypes at early stages. They also pointed out that sarcomas appear to be an almost negligible statistical threat to life in comparison to the “deadliest” cancer types, such as breast, lung, and colon cancers.

### 4.2. Cervical Cancer

Despite the application of the Papanicolaou test, cervical cancer remains the third most common cancer in women, second in mortality only after breast cancer. Again, early diagnosis represents the key strategy to reduce mortality; therefore, there is a constant need for novel biomarkers with high sensitivity and specificity.

Liu et al. [62] have previously shown the elevated levels of EV miRNA content in cervicovaginal lavage specimens in cervical cancer patients, implying a potential application of EV miRNAs in non-invasive screening for cervical cancers. Furthermore, they found that the expression of EV miRNA-21 and miRNA-146a in cervicovaginal lavage specimens of cervical cancer patients was significantly higher than in HPV-positive subjects and HPV-negative healthy subjects (in both *p* < 0.01), suggesting their potential contribution to cervical cancer development. More recently, Zhang et al. [63] analyzed EV lncRNA levels in a cohort of 30 cervical cancer patients; 30 cancer-free, HPV-positive subjects; and 30 HPV-negative healthy subjects. They showed that the expression of EV-HOTAIR and MALAT1 in the cervicovaginal lavage samples of cervical cancer patients was significantly higher than in HPV-positive subjects and HPV-negative, cancer-free subjects (*p* < 0.01 and *p* < 0.05, respectively). Conversely, the EV-MEG-3 level was significantly lower than in HPV-positive subjects and HPV-negative, cancer-free subjects (*p* < 0.01 and *p* < 0.05, respectively). In a different study, ATF1 and RAS were found to be significantly elevated in tumors of primary and recurrent cervical cancer mouse models. Moreover, they found that ATF1 and RAS were among the 5 most expressed genes detected in circulating EVs, proposing them as diagnostic markers for cervical cancers [64].

Honegger et al. [65] demonstrated that the expression of the E6/E7 oncogenes in HPV+ cancer cells can induce the upregulation of EV-miR-21-5p and the downregulation of 6 cervical-cancer-associated EV miRNAs (let7d-5p, miR-20a-5p, miR-378a-3p, miR-423-3p, miR-7-5p, and miR-92a-3p). Recently, miRNA-7, miRNA-99, miRNA-378, and the miRNA-17-92 miRNA families were found to be the most dysregulated EV miRNAs in HPV-associated cancers, particularly in cervical cancer [66]. EV-associated biomarkers investigated for cervical cancer diagnosis are reported in Table 2.

## 5. Renal Cancer

Renal cell carcinoma (RCC) is one of the most common malignant tumor of the urinary system, being clear cell RCC (ccRCC) the most common subtype of RCC, which accounts for 70–85% of the renal parenchymal cancers [6]. A few studies have investigated the role of urinary EVs as biomarkers in RCC. A RCC-specific signature including 10 up- or downregulated proteins (such as MMP-9, podocalyxin (PODXL), Dickkopf-related protein 4 (DKK4), carbonic anhydrase IX (CAIX) and ceruloplasmin) were found in the urinary EVs from RCC patients by proteomics [67]. In a different study, urinary EV transcriptomics showed a significant difference in mRNA content in ccRCC patients compared to healthy controls and patients with other RCC types: lower level of EV-GSTA1, CEBPA and PCBD1 mRNA content was proved specific for ccRCC. These data were further sustained by normalization of their EV expression one month after treatment [68].

Urinary EV lipidomics were also investigated by Del Boccio et al. [69], who demonstrated a differential lipid composition between RCC patients and healthy subjects, suggesting a relationship between the lipid compositions of urinary EVs and RCC.

In order to identify a specific biomarker that could distinguish between ccRCC patients and patients with other urinary cancers, Song et al. [70] evaluated urine EV miRNAs obtained from ccRCC patients, prostate and bladder cancer patients, and healthy controls. They identified miR-30c-5p as being significantly downregulated in ccRCC patients compared to normal individuals. Furthermore, no significant differences in the urine EV miR-30c-5p content were found between prostate and bladder cancer patients and healthy individuals. For the ccRCC diagnosis, the urinary EV-miR-30c-5p content displayed sensitivity and specificity corresponding to 68.57% and 100%, respectively, while the AUC was 0.8192 (95% confidence interval, *p* < 0.1). Butz et al. [71] showed that several EV-derived miRNAs used in combination can differentiate ccRCC patients from healthy controls and patients with benign lesions. In a recent study, Zhao et al. [72] collected serum and urine samples from 4 ccRCC patients before and after surgery and from controls. Western blot analysis of the EV protein content in urine showed that ccRCC was associated with increased expression and secretion of PTRF, which significantly decreased after surgery. PTRF was shown to play an important role in the formation and secretion of EV in malignant tumors and to be mainly regulated by the EGFR-Akt pathway. Interestingly, the expression of PTRF was reduced in both cells and EV surfaces by knocking down Shc1 and inhibiting the Akt pathway in vitro. This demonstrates that the EGFR pathway regulated by Shc1 is closely related to the EV release and cargo in ccRCC. Moreover, the authors suggested that urine EV enriched in PTRF can reflect the expression of Shc1, proposing it as a ccRCC biomarker.

EV isolation from clear cell renal cell carcinoma tissue is particularly difficult due to the intracellular lipids that give the white, “clear” appearance to the ccRCC cells. Based on this principle, recently Zieren et al. [73] first used NanoFCM on EVs from kidney (tumor) tissue, demonstrating its advantages over NanoSight; hence, they proposed this optimized protocol for biomarker discovery and EV biological studies in renal cancers.

Kurahashi et al. [30] focused on the RCC subtype TFE3 Xp11 translocation RCC (tRCC), which has been recently recognized as the cause of almost 42% of RCC in children and young adults [74]. The authors not only demonstrated the significantly increased expression of miR-204-5p in urinary EVs of 40-week-old tRCC Tg mice compared to controls, but also that its expression is increased in urinary EVs from 20-week-old Tg mice prior to the development of tRCC. Moreover, since its increase in the 20-week-old mice was similar to the 40-week-old Tg mice, miR-204-5p was proposed as a useful biomarker for early diagnosis of Xp11 tRCC patients. EV-associated biomarkers investigated for renal cancer diagnosis are reported in Table 3.

## 6. Bladder Cancer

Bladder cancer (BCa) is one of the five most frequent malignant tumor types in developed countries. Moreover, BCa is the second most common cancer among malignancies of the genitourinary tract [7]. BCa-derived EVs are released directly into the urine; therefore, urine is an excellent source for biomarker discovery in BCa. The concentration of CD63-positive urinary EVs is significantly elevated in BCa patients compared to healthy controls, demonstrating the potential application of EVs as disease biomarkers [75]; however, few studies have been performed and EVs are still far from being exploited as biomarker tools in BCa clinical practice [76].

Several studies have focused on the proteomic profiling of EVs released by BCa cell lines and urinary EVs from BCa patients [77,78] to allow the development of reference libraries for biomarker discovery approaches in patient samples [77,78]. These studies demonstrated that seven proteins (i.e., APOA1, CD5L, FGA, FGB, FGG, HPR, and HP) were differentially expressed when low- and high-grade BCa were compared. Furthermore, tumor-associated calcium signal transducer 2 (TACSTD2) was proposed as a diagnostic biomarker owing to its content in patients with BCa. Welton et al. [79] demonstrated a strong association of the proteomic profiling of EVs derived from the HT1376 bladder cancer cell line and showed elevated levels of CD36, CD44, 5T4, basigin, and CD73 in BCa. Lin et al. [80] applied MALDI-TOF spectrometry to demonstrate the enrichment of alpha 1-antitrypsin and histone H2B1K in urinary EVs. Based on the AUC value of 0.87, they proposed this EV enrichment for diagnostic and prognostic purposes, showing the sensitivity and specificity of the combination of the two peaks for detecting urothelial cancer (UC) of 62.70% and 87.59%, respectively. Periostin has also been considered as a potential BCa biomarker, since a high level was found in urinary EVs from BCa patients compared to healthy subjects. Furthermore, periostin-enriched EVs have been proven to increase aggressiveness, to promote progression, and to correlate with a poor clinical outcome [81,82].

Noncoding RNAs (e.g., lncRNAs, miRNAs, and mRNAs) in EVs are considered a promising class of biomarkers. In 2014, Perez et al. [83] performed differential gene expression profiling in urinary EVs from 3 BCa patients and 5 healthy subjects by using a whole transcriptome array, followed by PCR validation. They found EVs enriched in the polypeptide *N*-acetylgalactosaminyltransferase 1 (GALNT1) and ceramide synthase 2 (CERS2) mRNAs only in cancer patients, while finding EVs enriched in the tumor suppressors, ARHGEF39, and FOXO3 mRNAs only in controls. Given the number of samples analyzed, this may be considered as a pilot study providing a methodological approach to identify candidate mRNAs potentially useful for biomarker discovery in BCa.

Several studies have focused on the identification of diagnostic and prognostic miRNAs in BCa; however, different miRNAs have been identified [84]. Andreu et al. [85] applied qPCR to validate EV miRNAs selected by microarray analysis from 34 morning first urine samples collected from patients with BCa (18 high-grade and 16 low-grade) prior to surgery and from 9 healthy volunteers. They demonstrated that miR-375 was significantly lower in high-grade BCa patients compared to healthy volunteers, while miR-146a was found to be upregulated in low-grade BCa patients compared to high-grade patients. Matsuzaki et al. [86] analyzed miRNAs in urinary EV from 36 patients and 24 controls (donors for kidney transplantation, healthy volunteers, and postoperative patients of urothelial carcinoma). They identified the EV-miR-21-5p content as the most powerful biomarker for detecting urothelial carcinoma, displaying an AUC of 0.900 (the sensitivity and specificity were 75.0% and 95.8%, respectively). In a different study, the presence of miR-1224-3p, miR-135b, and miR-15b and the ratio of miR-126 to miR-152 in urinary pellets were shown to correlate with the diagnosis of BCa [87]. Armstrong et al. [88] also identified several upregulated miRNAs (miR-4454, miR-720, miR-21, miR-205-5p, and miR-200c-3p) in urinary EVs from BCa patients. More recently, Amuran et al. [89] suggested that urine EV miR-19b1-5p, 136-3p, and 139-5p contents and urinary APE1/Ref1, BLCA-4, and CRK concentrations could be promising candidates for BCa diagnosis. Moreover, they proposed a combo panel that differentiates BCa patients from healthy controls with 80% sensitivity and 88% specificity (AUC = 0.899) and differentiates low-risk patients from controls with 93% sensitivity and 95.5% specificity (AUC = 0.976).

Berrondo et al. [90] focused on long non-coding RNA (lncRNA) in BCa EVs. They demonstrated that HOTAIR, HOXA cluster antisense RNA 2 (HOXA-AS2), metastasis-associated lung adenocarcinoma transcript 1 (MALAT1), the mRNAs for SRY-box 2 (SOX2), and POU class 5 homeobox 1 (POU5F1) were selectively enriched in urinary EVs from eight patients with high-grade muscle-invasive urothelial BCa compared to urinary EVs from healthy volunteers. More recently, the diagnostic value of EV-H19 content for distinguishing BCa patients from benign tumors and healthy individuals was analyzed by Wang et al. [91]. The ROC curve analysis showed an AUC of 0.851 with a sensitivity and specificity reaching 74.07% and 78.08%, respectively. Zhang et al. [92] identified a panel of three lncRNAs (PCAT-1, UBC1, and SNHG16) characterized by high BCa diagnostic accuracy with AUC values of 0.857 and 0.826 in training and validation sets, respectively. This was a significantly higher value than that of urine cytology [92]. Similarly, a panel consisting of three differently expressed urinary EV lncRNAs (MALAT1, PCAT-1, and SPRY4-IT1) was proposed by Zhan et al. [29] for BCa diagnosis, showing an AUC of 0.854 for the training set and an AUC of 0.813 in the validation set. Again, these values were significantly higher than for urine cytology.

Urinary EV-DNA content could be considered as an alternative liquid biopsy source to identify genetic alterations in BCa, as well as in other malignancies. A pilot study by Lee et al. [93] included 9 patients who underwent surgery for BCa. They found that urinary cfDNA and EV-DNA matched the genomic profiling of tumor samples, firstly identifying the somatic mutations and copy number variations (CNV) in BCa. EV-associated biomarkers investigated for bladder and urothelial cancer diagnosis are reported in Table 4.

## 7. Prostate Cancer

Prostate cancer (PC) is the most common solid malignancy and the second cause of mortality in men in Western countries, due to the high rate of deferred diagnosis [5]. The most used biomarker, PSA, does not accurately discriminate between aggressive cancers and indolent, more benign lesions such as benign prostatic hyperplasia (BPH) and prostatitis. EV derived from serum, plasma, urine, and seminal fluid are summarized here as proposed biomarkers.

Several EV isolation methods have been applied in PC [94]. Even though consensus has not yet emerged on specific EV markers [12,95], several proteins alone or in combination have been proposed as EV biomarkers for PC.

Circulating EVs harbor specific PC proteins. PTEN has been detected in patients with PC but not in healthy subjects [96]. Minciacchi et al. [97] demonstrated that cytokeratin 18 (CK18) is significantly enriched in circulating EVs from PC patients, in line with its expression in tissue samples. Kawakami et al. [98] showed that the EV GGT content is significantly higher in PC patients compared with BPH individuals and suggested the feasibility of this marker to discriminate patients with PC and BPH who display close PSA levels. Indeed, the AUC for serum EV GGT activity was found to be higher than for serum GGT activity and serum PSA concentration. Khan et al. [99] suggested the EV survivin content as a promising biomarker for patients with high PSA with or without tumors, reporting that survivin in circulating EV shows higher expression in patients with PC compared to BPH and healthy controls. Park et al. [100] found that PSMA- and CD63-positive circulating EVs were differentially expressed in BPH and PC patients. In a recent study, Øverbye et al. [101] found that TM256 in urine EVs was able to discriminate PC patients (n = 16) from controls (n = 15) with a sensitivity of 94%. The sensitivity reached 100% when TM256 was analyzed in combination with the autophagy inhibitor late endosomal–lysosomal adaptor, MAPK, and MTOR activator 1 (LAMTOR1). A different panel consisting of flotillin 2 and parkinsonism-associated deglycase (PARK7) enriched in urine EVs from PC patients was suggested by Wang et al. [102]. Similarly, the overexpressed level of fatty-acid-binding protein 5 (FABP5) in EVs from PC has been associated with the Gleason score [103]. Lu et al. [104] demonstrated that δ-catenin, caveolin-1, and CD59 were positive in urine EVs of PC patients, proposing them as PC biomarkers. Principe et al. [105] identified 877 proteins derived from the prostatic secretions in urine, 14 of which were most-readily detectable in EVs (ACPP, LTF, DDP4, TGM4, MME, PSA, SEMG1, AZGP1, ANPEP, G3BP, PSMA, TMPRSS2, FASN, LGALS3, PSCA, KLK2, KLK11, TIMP1, ANXA2, CLSTN1, FASN, FLNC, FOLH1). Increased levels of α1-integrin and β1-integrin were found in urine EVs of patients with metastatic PC compared with patients with BPH [106]. Similarly, Sequeiros et al. [107] distinguished PC and BPH by combining EV contents of adseverin and transglutaminase-4.

Seminal fluid and prostatic secretions, which contain considerable amounts of EVs, represent ideal fluids for EV-derived biomarker discovery; however, the major drawbacks are the limited studies and the lack of a systematic, high-quality biobanking system for proximal prostate fluids [105,108,109,110].

Several differentially expressed proteins in EV from PC cell lines have been also identified (PDCD6IP, XPO-1, ENO1, CDCP1, CD151, CD147) [111,112,113], although the lack of expression in urine-derived EVs has limited the use of PC cell lines for biomarker discovery [114].

The EV PSA content has also displayed great potential as a more reliable marker than serum PSA. Logozzi et al. [115] recently showed that PC patients had four-fold levels of circulating EVs expressing both CD81 and PSA compared to BPH patients and healthy controls. Similarly, Øverbye et al. [101] showed that the PC protein markers (PSA, FOLH1/PMSA, TGM4, and TMPRSS) are highly expressed in EVs derived from PC patients compared to healthy subjects.

Despite prostasomes (EVs secreted by epithelial cells in the prostate gland) being considered the most accurate sources for proteomic or transcriptomic biomarker discovery in PC, to date only one study has reported that metastatic PC tissues release EVs, displaying altered annexins A1, A3, A5, and dimethylarginine dimethylaminohydrolase 1 expression [116]. A highly specific and sensitive method named the proximity ligation assay has been applied to detect elevated levels of prostasomes in blood samples from patients with PC before radical prostatectomy [37].

The study of glycan sugar groups, which are closely associated with the development and progression of PC, holds promise for the stratification of PC patients [117]. As EVs are enriched in specific glycans, glycomics can be used to study EV surface glycans or glycoproteins to improve PC diagnosis. Glycan profiling of urinary EVs derived from prostatic secretions indicated that changes in glycosylation of N-linked glycoproteins, such as an increase in larger tetra-antennary glycans, might reflect the clinical status of PC patients; however, no conclusions can be drawn for these limited pilot analyses on pooled samples [108,109,110,111,112,113,114,115,116,117]. Some data have been reported on the role of the cancer biology of EV glycosylation, particularly in PC. A better understanding of glycans and glycoproteins associated with EVs may provide new avenues for PC diagnosis and monitoring of progression [94].

Several studies on protein cargo have identified potential EV biomarkers; however, these data should be confirmed in larger cohorts of patients to move towards the development of EV-based non-invasive diagnostic tools for personalized medicine [97].

Recently, the meta-analysis by Yang et al. [118] confirmed that plasma EV miRNAs have high diagnostic value for PC patients. Hessvik et al. [119] identified 36 EV miRNAs as candidate biomarkers for PC in clinical studies.

PC-associated transcripts such as the lncRNA PCA3 and the TMPRSS2-ERG fusion mRNA were detected in urinary EVs in PC patients [97,120,121]. Nilsson et al. [120] analyzed the RNAs in EVs from urine of nine PC patients and demonstrated that urinary EVs are enriched in TMPRSS2:ERG gene fusion. This gene fusion results from a chromosomal rearrangement of ERG to the androgen-responsive gene TMPRSS2 and prostate cancer antigen 3 (PCA3). PCA3 is a lncRNA and is considered one of the most specific PC biomarkers. Donovan et al. [122] recently developed a patient score (EXO106) based on mRNA levels of PCA-3, ERG, and SPDEF in urine-derived EVs, which predicts both PC and high-grade disease (Gleason score 7 or higher), with an AUC corresponding to 0.764. This score allows the high-grade PC diagnosis of ‘grey zone’ patients based on serum PSA levels. In a follow-up study, the score provided by assaying PCA-3, ERG, and SPDEF gene expression in urine EVs (ExoDx Prostate IntelliScore) outperformed standard of care values, discriminating PC patients with ≥GS7 from GS6 or with a negative biopsy (AUC = 0.73). In a large validation study, this score improved the discrimination among high-grade, low-grade, and benign disease compared with the standard of care [121]. In a different study, Isin et al. [31] evaluated the EV contents of two tumor-suppressive lncRNAs, named GAS5 and TP53COR, in urine samples after digital rectal examination of 30 patients with PC and 49 BPH patients. TP53COR1 was more expressed in PC patients than in patients with BPH and the sensitivity and specificity of TP53COR1 to predict PC corresponded to 67% and 63%, respectively, while 94% specificity was reported when TP53COR1 was considered in combination with PSA. Foj et al. [123] analyzed five miRNAs commonly deregulated in PC (tumor tissues, serum or plasma, or EVs from freshly voided urine samples) and miR-375, miR-21, and let-7c were found significantly to be upregulated in the PC group compared with the healthy group, with AUC values corresponding to 0.799, 0.713, and 0.679, respectively. Furthermore, they identified a panel of miRNAs from a urinary pellet, mainly consisting of miR-21 and miR-375, able to discriminate between healthy individuals and PC patients (AUC 0.872); however, the small sample size was the most relevant limitation of the study.

The role of miR-375 was also confirmed by Endzeliņš et al. [124]. Rodriguez et al. [125] reported that miR-196a-5p and miR501-3p are significantly decreased in urinary EVs from PC patients, with AUC values corresponding to 0.73 and 0.69, respectively. Korzeniewski et al. [126] identified three miRNAs (miR-483-5p, miR-1275, miR-1290) as the most abundant miRNAs released by PC cells. Furthermore, miR-483-5p alone, as well as in combination with miR-1275 and miR-1290, was able to significantly discriminate PC patients with biopsy-proven tumor mass from patients with microscopic tumors; however, these miRNAs failed to show advantages over conventional methods. Samsonov et al. [127] proposed the upregulation of miR-21, miR-574, and miR-141 in urinary EVs as diagnostic markers based on the AUC (0.86). Recently, Kohaar et al. [128] used a 2-gene panel (PCA3, PCGEM1) to improve the prediction of high-grade cancer at diagnosis compared to standard of care variables in a racially diverse patient cohort (AUC of 0.88 vs 0.80, respectively). Different urinary EV miRNAs potentially diagnostic for PC include miR-19, miR-145, and miR-2909 [102,129,130,131].

Little is known about the potential application of EV lncRNAs as circulating diagnostic markers for PC. Isin et al. [31] suggested lncRNA-p21, a suppressor of p53 signaling, as a marker, whose level in urinary EVs could distinguish PC patients from those with the benign disease.

Bryant et al. [132] showed that miR-107 and miR-574-3p were increased in the circulating EVs of men with non-metastatic PC compared with healthy individuals. The authors also demonstrated that both miR-141 and miR-375 were upregulated in patients with recurrent metastatic PC compared to patients with the non-recurring disease (estimated AUC = 0.8). The role of miR-141 as a PC oncomiR was further supported by the analysis of 20 patients with PC, 20 patients with BPH, and 20 healthy volunteers, in whom Hao et al. [133] demonstrated that circulating EV-miR-141 levels were significantly higher in PC patients than in BPH patients or healthy controls. Endzeliņš et al. [124] also suggested that miR-200 and miR-21 enriched in circulating EVs could differentiate PC from BPH patients, with AUCs corresponding to 0.68 and 0.67, respectively. Other potentially diagnostic circulating markers for PC include the splice variant of the AGR2 transcript and miR-1246 [134,135].

PC DNA aberrations, such as PTEN and TP53 mutations, can be detected by analyzing urine EV DNA content. The profiling of EV enriched in mutated DNA in combination with the EV-RNA expression provides a comprehensive map of intra-EV changes in PC using urine-based liquid biopsies [136].

Lipid EV composition was reported as a potential biomarker for the development of PC. Several lipid classes, such as diacylglycerol (DAG) and triacylglycerol (TAG) species, are differentially enriched in urinary EVs from PC patients and healthy controls, and differences in the EV molecular lipid species from non-tumorigenic, tumorigenic, and metastatic prostate cells have been proposed for diagnostic purposes [137,138].

The analysis of small metabolites can potentially reveal dynamic changes in the metabolism downstream of genetic and proteomic regulation. Metabolomic profiling of urinary EVs is considered feasible to identify disease profiles, which cannot be revealed by conventional urine analyses. For example, the levels of adenosine, glucuronate, isobutyryl-L-carnitine, and D-ribose 5-phosphate were significantly lower in pre-prostatectomy urine-derived EVs as compared to post-prostatectomy and control samples [139].

In a recent pilot study, Rikkert et al. [140] tried to determine whether metastatic castration-resistant PC patients could be discriminated from healthy controls based on the presence of EV subtypes in plasma or urine samples, using flow cytometry (FCM) and surface plasmon resonance imaging (SPRi); however, a significant difference between patients and controls was only found for the lactadherin+ particle and the EV plasma concentration. EV-associated biomarkers investigated for prostate cancer diagnosis are reported in Table 5.

## 8. Ovarian Cancer

Despite the development of new therapeutic approaches (pharmacological and surgical), the mortality rate for ovarian cancer (OC) patients still remains one of the highest among females, with a 5-year relative survival rate corresponding to 50%. Less than 20% of the OCs are diagnosed at the initial stage. This gives a 90% 5-year related survival rate; therefore, early diagnosis is crucial and EV-related biomarkers are emerging as promising tools [3,141].

In 2019, Barnabas et al. [142] analyzed EVs derived from utero-tubal lavage (UtL) of 49 women with high-grade serous ovarian cancer (HGSOC) and compared them to 121 healthy women. They suggested a panel of 9 differentially expressed proteins (SERPINB5, S100A14, MYH11, CLCA4, S100A2, IVL, CD109, NNMT, and ENPP3) able to diagnose HGSOC with a sensitivity and specificity corresponding to 70% and 76.2%, respectively. In addition, EpCAM was found to be increased in EVs recovered from body fluids of patients with epithelial ovarian cancer (EOC) [143,144,145,146,147].

The role of EpCAM as a biomarker was further confirmed by Zhang et al. [148], who performed a quantitative proteomic analysis of serum EVs from 10 EOC patients and 10 healthy controls. Indeed, they demonstrated that EVs are enriched in EpCAM, C1q, ApoE, and plasminogen and depleted of serpin C1. ApoE multiplexed with EpCAM, Plg, serpin C1, and C1q provided optimal diagnostic performance for EOC, with an AUC corresponding to 0.913. Liang et al. [149] identified protein signatures in EOC-derived EVs and tissues, including EpCAM, PCNA, TUBB3, EGFR, ApoE, CLDN3, FASN, ERBB2, and L1CAM, which were proposed as diagnostic OC markers. On the other hand, in a different study, EVs derived from human ovarian epithelial cells (HOSEPiC) were compared to three OC cell lines (OVCAR3, IGROV1, and ES-2). The authors showed low EpCAM detection (<20%), suggesting that EpCAM should not be considered a proper OC marker in the early stages [150].

In conclusion, despite some results having suggested the possibility of combining EpCAM with CD24 to detect OCOvCa-derived EVs, large-scale clinical trials are needed to verify this hypothesis [151].

In 2017, Stope et al. [152] proposed the heat shock protein 27 (HSP27) as a potential biomarker, since it was increased in EOC patient EVs. Zhang et al. [153] compared circulating EV protein contents from EOC patients and healthy controls and identified four promising markers for diagnosis and prognosis (LBP, FGG, FGA, and GSN). More recently, Dorayappan et al. [154] reported 3 proteins that were significantly elevated in serum EVs (HGF, STAT3, and IL6) as valuable candidates for the early detection of OC. Peng et al. [155] selected the AKT1, FAM49B, CLIC4, LTF, SNX3, TUBB3, URP2, and MAP II in circulating EVs as potential biomarkers for EOC diagnosis. Other studies have suggested claudin-4 and TrkB as potential EV biomarkers [156,157]. In a recent study by Cheng et al. [158], collagen type V alpha 2 chain (COL5A2) and lipoprotein lipase (LPL) were found to be significantly higher in ovarian cancer cell line-derived EVs than in ovarian surface epithelial cells (HOSEPiC) (*p* < 0.05). In a different study, soluble E-cadherin (sE-cad) released with EVs into the ascitic fluid was proposed as a potential biomarker able to distinguish OC from benign disease [159].

In 2008, Taylor et al. [144] suggested a specific combination of EV miRNAs, including miR-21, miR-141, miR-200a, miR-200c, miR-200b, miR-203, miR-205, and miR-214, as surrogate OC diagnostic markers. More recently, Pan et al. [160] demonstrated statistically significant overexpression of miR-23a, miR-92a, miR-21, miR-100, and miR-200b and downregulation of miR-320, miR-16, miR-93, miR-126, and miR-223 in circulating EVs from EOC patients when compared with healthy women. The authors focused on miR-21, miR-100, and miR-200b, since they were apparently able to discriminate between EOC patients and healthy women, with sensitivity levels of 61%, 62%, and 64% and specificity levels of 82%, 73%, and 86% respectively. Importantly, the combination of those values did not increase the detection power. Meng et al. [161] showed that circulating EV miR-373 (*p* = 0.0001), miR-200a (*p* = 0.0001), miR-200b (*p* = 0.0001), and miR-200c (*p* = 0.028) contents were significantly higher in EOC patients than in healthy women. Furthermore, the authors identified a panel consisting of EV miR-200a, miR-200b, and miR-200c that could discriminate between EOC patients and patients with benign ovarian diseases, with a sensitivity of 88% and a specificity of 90%. Importantly, miR-200a alone differed between these patient cohorts, with a sensitivity of 84% and a specificity of 90%, while miR-200a was found to be elevated in mucinous and serous carcinomas compared to other EOC subtypes [162]. In a different study, Kim et al. [163] demonstrated a sensitivity of 91.6% for the circulating EV miR-145 content and a specificity > 90.0% for EV-miR-200c compared with CA125 for a preoperative diagnosis of OC; however, further studies are needed to elucidate the potential differences between miR-200 family and EV miR-200 family contents for both OC diagnosis and prognosis [164].

More recently, Yamamoto et al. [165] identified five mRNAs (CA11, MEDAG, LAMA4, SPINT2, NANOG) and six miRNAs (let-7b, miR23b, miR29a, miR30d, miR205, miR720) that were differentially expressed in cancer ascites and peritoneal fluids from benign patients. Furthermore, a combination of LAMA4, CA11, MEDAG, NANOG, SPINT2, let-7b, miR23b, and miR29a was selected based on their diagnostic performances; however, reliable conclusions could not be drawn due to the small sample size. Similarly, the overexpression of miR-99a-5p in serum-derived EVs of 62 OC patients compared with 20 healthy controls along with its decrease after de-bulking surgery (*p* = 0.003) was shown in a different study by Yoshimura et al. [166]. The diagnostic performance for EOC detection showed 85% sensitivity and 75% sensitivity. Interestingly, these data changed according to the tumor histology (sensitivity and specificity of 84% and 40%, respectively, for serous EOC; 33% and 91%, respectively, for clear-cell EOC; 67% and 82%, respectively, for endometrioid EOC; 33% and 91%, respectively, for mucinous EOC). These data strongly suggest the need for more tailored diagnostic approaches. Finally, when compared with CA125, miR-99a-5p showed improved sensitivity (87 vs. 54) with comparable specificity (73 vs. 75) for the early EOC detection, justifying further investigation of its clinical role. Wang et al. [167] identified five differentially expressed miRNAs (miR-205-5p, miR-145-5p, miR-10a-5p, miR-346, and miR-328-3p) in circulating EVs from EOC patients when compared with a control group, pointing out the higher diagnostic accuracy (AUC: 0.760; 95% CI: 0.691–0.828) when the five miRNAs were combined. Su et al. [168] also highlighted the diagnostic value of high circulating EV-miR-375 and miR-1307 contents.

Masaki Kobayashi et al. [169] showed that miR-1290 was highly expressed in circulating EVs from 70 EOC patients, although when compared with healthy controls, no statistically significant differences were detected (*p* = 0.89).

Critical evaluation of the published studies indicates that the accuracy of single miRNAs as diagnostic marker is inadequate due to histological diversity and individual differences, and strongly suggests that a panel of EV biomarkers may be more useful [170,171]; however, even if the data appear promising, several limitations such as the small sample size with no external validation and the lack of studies evaluating the role of the mentioned biomarkers integrated in clinical algorithms (including symptom evaluation, CA125 dosage, and ultrasound findings) prevent us from drawing definitive conclusions [172].

In addition to various protein and miRNA markers, several different biomolecules have been reported as critical for OC diagnosis [173]. They include phosphatidylserine EVs [174,175] and EV mitochondrial DNA (mtDNA) copy numbers [176]. Cheng et al. [158] recently identified 1433 proteins and 1227 lipid species in EVs derived from ovarian cancer cells (SKOV-3) and ovarian surface epithelial cells (HOSEPiC). Among the 110 lipids differentially expressed between each group, the highest significance was detected for PG (34:1)-H and ChE(18:2) + NH4. Furthermore, since some lipid species showed species specificity, the potential application of EV lipid species as cancer biomarkers has been proposed. EV-associated biomarkers investigated for ovarian cancer diagnosis are reported in Table 6.

## 9. Drawbacks for Clinical Application of EVs

Although few studies have investigated the economical and pragmatic feasibility of the diagnostic application of EVs, the current literature gives a picture of their clinical application as promising. In 2018, the ISEV provided a guide to standardize the collection, isolation, and analysis of EVs using different biofluids [12]. For the types of cancers discussed herein, blood (plasma or serum) and urine have been proposed as the most suitable EV sources for biomarker discovery; however, when collecting blood, particular attention should be devoted to the choice of anticoagulants. Heparin-based anticoagulation is discouraged, since it can inhibit the PCR reaction, providing false negative results. On the other hand, citrate and ethylenediaminetetraacetic acid (EDTA) can potentially decrease EV concentrations in clinical samples [177,178]. This implies that the choice of the anticoagulant should be ideally based on the downstream EV analysis. Moreover, dealing with blood-derived EVs, circadian rhythms should also be considered for the expression of specific EV surface markers [177]; however, the impact of circadian rhythms on the diagnostic accuracy is still debated. Urine collection is easy and non-invasive, making it the most promising body fluid for liquid biopsies. To avoid uromodulin-mediated EV capture, diluting agents have been proposed [179]. Moreover, attention should be also directed to avoid urine bacterial contamination. Overall, these precautions are not specific for EV isolation, being similar for the daily handling of biological fluids, meaning they cannot likely be considered as additional economical or time-consuming issues. So far, studies addressing time consumption for EV isolation are lacking; however, it has been demonstrated that the timing of EV isolation can match with the workload of a diagnostic laboratory. Saenz-Cuesta et al. [25] proposed a protocol for hospital-based EV isolation by comparing different isolation methods, all of which were found to be suitable.

In addition, Konoshenko et al. [17] proposed a precipitation-based isolation protocol from blood and urine in healthy and prostate cancer patients requiring no more than 110 min for sample preparation and EV isolation. Considering that one of the most commonly ordered diagnostic tests, the blood culture test, generally requires a turn-around time (TAT) of 2 days, we are confident that EVs can become good candidates to enter into clinical practice as diagnostic tools [180].

## 10. Conclusions

Cancer is one of the major global health problems from both clinical and economic standpoints. Advances in modern medicine, along with the progressively aging global population, suggest that cancer care will become even more challenging in the future. The improvement of therapeutic cancer approaches driven by basic and clinical research is crucial to move the field ahead. Similarly, innovative and improved diagnostic strategies are needed in order to ameliorate the prognosis of cancer patients. As emerged from data provided in this review, EVs could likely be used as diagnostic tools in innovation processes. Given their cellular origin, cancer-derived EVs, which carry a huge variety of biomarkers mirroring the contents of malignant cells, can be recovered through non-invasive, affordable, and time-saving techniques (e.g., venous blood and urine sampling); therefore, it is tempting to speculate that EVs represent one of the most promising cancer derivatives in oncology. This is further supported by the observation that several EV-associated biomarkers are shared by many tumors (Table 7).

It is known that miR-21 is one of the most common onco-miRNAs, whose role in carcinogenesis has been progressively recognized [181]. Furthermore, miR-21 has been involved in many cancer-related biological processes, such as proliferation, invasion, and metastasis formation, and has been found to be upregulated in many types of cancer. From a diagnostic standpoint, miR-21 dysregulation in EVs shows high consistency among tumors. Indeed, it was generally found to be upregulated rather than downregulated in EVs from cancer patients [46,62,86,88,118,123,124,144,160]. A different example is provided by the miR-200 family, which is mainly involved in the regulation of tumor development and proliferation [182,183]; however, despite their anti-tumor properties, the miR-200 family members were found to be consistently overexpressed in EVs derived from prostate, ovarian, and bladder cancers. The different functions and targets in discrete tumors as well as the different functions in intracellular compartments and EV cargo may explain these observations. Additional data are required to address this issue. On the other hand, EV-related biomarkers have been described as highly tumor-specific, reflecting the peculiarities of their tissues of origin; EVs enriched in TMPRSS2:ERG fusion transcript in prostate cancer are an example [120]. Nevertheless, it should be kept in mind that biomarkers shared by many different types of cancer tend to lose specificity, making their diagnostic application (e.g., screening of asymptomatic patients) at early stages inconsistent. On the other hand, the same EV biomarkers could be valuable in patients with a high index of suspicion for a specific cancer (e.g., in combination with radiological investigation) or once a definitive diagnosis is obtained during the follow-up.

The research on EVs as diagnostic tools should, therefore, be encouraged. The combined efforts from clinical radiology and biochemistry approaches would bring pioneering bedside diagnostic multimodal protocols to patients. The numerous clinical trials currently ongoing (https://clinicaltrials.gov/ct2/results?cond=cancer+&term=exosomes%2C+diagnosis&cntry=&state=&city=&dist= (accessed on 24 June 2021)), in addition to confirming the diagnostic potential of EVs, may provide the rational to transfer their application to day-to-day clinical practice.

## Figures and Tables

**Table 1:** EV-associated biomarkers in breast cancer.

mRNAs	Up/Downregulation	Fold-Change	References
Lactate Dehydrogenase C	Upregulated	N/A	[45]
**miRNAs**			
miR-21	Upregulated	16.51	[46]
miR-1246	Upregulated	8.72	[46]
miR-106a-3p	Downregulated	2.55	[47]
miR-106a-5p	Upregulated	3.59	[47]
miR-20b-5p	Upregulated	11.25	[47]
miR-92a-3p	Downregulated	5.54	[47]
miR-92a-2-5p	Upregulated	2.35	[47]
miR-19b-3p	Upregulated	10.18	[47]
miR-424	Upregulated	N/A	[33]
miR-423	Downregulated	N/A	[33]
miR-660	Downregulated	N/A	[33]
Let7-i	Downregulated	N/A	[33]
miR-142-5p	Upregulated	N/A	[48]
miR-320a	Upregulated	−2.03	[48]
miR-4433b-5p	Upregulated	N/A	[48]
miR-101	Upregulated	N/A	[49]
miR-372	Upregulated	N/A	[49]
miR-373	Upregulated	N/A	[49]
**Proteins**			
EpCAM	Upregulated Upregulated	N/A	[36] [57]
Fibronectin (FN)	Upregulated	N/A	[51]
LDH-C4	Upregulated	N/A	[45]
CD47	Expressed	N/A	[52]
HER2	Expressed Expressed	N/A	[36] [57]

N/A: Not Available.

**Table 2:** EV-associated biomarkers in cervical cancer.

miRNAs	Up/Downregulation	Fold-Change	References
miR-21	Upregulated	N/A	[61]
miR-146a	Upregulated	N/A	[61]
miR-20a-5p	Downregulated	1.5	[65]
miR-92a-3p	Downregulated	1.5	[65]
miR-423-3p	Downregulated	1.5	[65]
miR-378a-3p	Downregulated	1.5	[65]
miR-7d-5p	Downregulated	1.5	[65]
**lncRNA**			
HOTAIR	Upregulated	≃5	[63]
MALAT1	Upregulated	≃3.5	[63]
MEG-3	Downregulated	≃3	[63]
**Proteins**			
ATF1	Upregulated	N/A	[64]
RAS	Upregulated	N/A	[64]

N/A: Not Available.

**Table 3:** EV-associated biomarkers in renal cell carcinoma.

mRNAs	Up/Downregulation	Fold-Change	References
GSTA1	Downregulated	−1.7	[68]
CEBPA	Downregulated	−1.89	[68]
PCBD1	Downregulated	−2	[68]
**miRNAs**			
miR-30c-5p	Downregulated	−6.39	[70]
miR-204-5p	Upregulated	N/A	[30]
**Proteins**			
PTRF	Upregulated	N/A	[72]
MMP-9	Upregulated	N/A	[67]
PODXL	Upregulated	N/A	[67]
DKK4	Upregulated	N/A	[67]
CAIX	Upregulated	N/A	[67]
Ceruloplasmin	Upregulated	N/A	[67]

N/A: Not Available.

**Table 4:** EV-associated biomarkers used for bladder cancer diagnosis.

mRNAs	Up/Downregulation	Fold-Change	References
GALNT1	Upregulated	−1.1	[83]
CERS2	Upregulated	N/A	[83]
ARHGEF39	Downregulated	N/A	[83]
FOXO3	Downregulated	1.1	[83]
**miRNAs**			
miR-21	Upregulated	N/A	[88]
miR-146a	Upregulated	11.3	[85]
miR-126	Ratio	N/A	[87]
miR-19b1-5p	Upregulated	N/A	[89]
miR-1224-3p	Downregulated	N/A	[87]
miR-135b	Downregulated	N/A	[87]
miR-15b	Downregulated	N/A	[87]
miR-152	Ratio	N/A	[87]
miR-4454	Upregulated	N/A	[88]
miR-21-5p	Upregulated	3.76	[86]
miR-720	Upregulated	N/A	[88]
miR-375	Upregulated	−3.3	[85]
miR-205-5p	Upregulated	N/A	[88]
miR-200c-3p	Upregulated	N/A	[88]
miR-136-3p	Expressed	N/A	[85]
miR-139-3p	Downregulated	N/A	[85]
**lncRNAs**			
MALAT1	Upregulated Upregulated	N/A N/A	[29] [90]
HOTAIR	Upregulated	≃10	[90]
HOXA-AS2	Upregulated	≃9	[90]
SOX2	Upregulated	N/A	[90]
POU5F1	Upregulated	N/A	[90]
H19	Upregulated	N/A	[91]
PCAT-1	Upregulated	N/A	[92]
UBC1	Upregulated	N/A	[92]
SNHG16	Upregulated	N/A	[92]
SPRY4-IT1	Upregulated	N/A	[92]
**Proteins**			
APOA1	Upregulated	N/A	[77]
CD5L	Upregulated	N/A	[77]
FGA	Upregulated	N/A	[77]
HPR	Upregulated	N/A	[77]
HP	Upregulated	N/A	[77]
TACSTD2	Upregulated	8.02	[77]
CD36	Upregulated	N/A	[79]
CD44	Upregulated	N/A	[79]
5T4	Upregulated	N/A	[79]
CD73	Upregulated	N/A	[79]
BSG	Upregulated	N/A	[79]
Alpha 1-antitrypsin	Upregulated	N/A	[80]
H2B1K	Upregulated	N/A	[80]
Periostin	Upregulated	N/A	[82]

N/A: Not Available.

**Table 5:** EV-associated biomarkers of prostate cancer.

mRNAs	Up/Downregulation	Fold-Change	References
TMPRSS2:ERG	Upregulated	N/A	[120]
ERG	Expressed	N/A	[122]
SPDEF	Expressed	N/A	[122]
PCA3	Expressed Upregulated	N/A N/A	[122] [128]
PCGEM1	Downregulated	N/A	[128]
AGR2	Upregulated	N/A	[134]
**miRNAs**			
miR-21	Upregulated Upregulated Upregulated	N/A N/A N/A	[123] [118] [124]
miR-145	Upregulated	N/A	[130]
miR-1246	Upregulated	30	[135]
miR-196a-5p	Downregulated	−2.375	[125]
miR-483-5p	Upregulated	23.59	[126]
miR-19	Upregulated	2.94	[129]
Let7-c	Upregulated	N/A	[123]
miR-574	Upregulated	3.90 (ratio)	[127]
miR-574-3p	Upregulated		[132]
miR-375	Upregulated Upregulated	N/A N/A	[123] [124]
miR-200	Upregulated	N/A	[124]
miR-501-3p	Downregulated	−7.315	[125]
miR-1275	Upregulated	22.81	[126]
miR-1290	Upregulated	4.9	[126]
miR-141	Upregulated Upregulated	11.7 (ratio) 3.85	[127] [133]
miR-2909	Upregulated	N/A	[131]
miR-107	Upregulated	11.26	[132]
**lncRNA**			
PCA3	Upregulated	N/A	[120]
TP53COR	Upregulated	N/A	[31]
P21	Upregulated	N/A	[31]
**Proteins**			
AZGP1	Expressed	N/A	[96]
CK18	Upregulated	N/A	[107]
GGT	Upregulated Expressed	N/A N/A	[98] [105]
CD63	Upregulated	N/A	[100]
TM256	Expressed	N/A	[101]
LAMTOR1	Expressed	N/A	[101]
Flotillin	Upregulated	N/A	[102]
PARK7	Upregulated	N/A	[102]
FABP5	Upregulated	2.31	[103]
D-catenin	Expressed	N/A	[104]
Caveolin-1	Expressed	N/A	[104]
CD59	Expressed	N/A	[104]
ACPP	Expressed	N/A	[105]
LTF	Expressed	N/A	[102]
DDP4	Expressed	N/A	[102]
TGM4	Expressed Upregulated	N/A N/A	[102] [98]
MME	Expressed	N/A	[102]
PSA	Expressed Upregulated Upregulated	N/A N/A	[102] [112] [98]
SEMG1	Expressed	N/A	[102]
ANPEP	Expressed	N/A	[102]
G3BP	Expressed	N/A	[102]
TMPRSS	Upregulated	N/A	[98]
TMPRSS2	Expressed	N/A	[102]
PASN	Expressed	N/A	[102]
LGALS3	Expressed	N/A	[102]
PSCA	Expressed	N/A	[102]
KLK2	Expressed	N/A	[102]
KLK11	Expressed	N/A	[102]
TIMP1	Expressed	N/A	[102]
ANXA2	Expressed	N/A	[102]
CLSTN1	Expressed	N/A	[102]
FASN	Expressed	N/A	[102]
FLNC	Expressed	N/A	[102]
FOLH1	Expressed	N/A	[102]
FOLH1/PMSA	Upregulated	N/A	[98]
A3-integrin	Upregulated	1–3.5	[103]
B1-integrin	Upregulated	1.5–3.5	[103]
Adseverin	Upregulated	1.34	[104]
Transglutaminase-4	Downregulated	0.6	[104]
Annexin A1	Altered expression	N/A	[113]
Annexin A3	Altered expression	N/A	[113]
Annexin A5	Altered expression	N/A	[113]
DDAH I	Altered expression	N/A	[113]
**DNA**			
PTEN	Mutated	N/A	[133]
TP53	Mutated	N/A	[133]
**Metabolites**			
Adenosine	Downregulated	N/A	[136]
Glucuronate	Downregulated	−22	[136]
Isobutyril-L-carnitine	Downregulated	N/A	[136]
D-ribose 5P	Downregulated	N/A	[136]

N/A: Not Available.

**Table 6:** EV-associated biomarkers investigated for ovarian cancer diagnosis.

miRNAs	Up/Downregulation	Fold-Change	References
miR-21	Upregulated Upregulated	N/A 2.3	[144] [160]
miR-145	Upregulated	47.7	[163]
miR-145-5p	Upregulated	5.10	[167]
miR-126	Downregulated	0.6	[160]
miR-92a	Upregulated	1.2	[160]
Let7-b	Downregulated	0.01–0.21 *	[165]
miR-320	Downregulated	1.5	[160]
miR-375	Upregulated	N/A	[168]
miR-720	Downregulated	0.01–0.21 *	[165]
miR-205	Upregulated Downregulated	N/A 0.01–0.21 *	[144] [165]
miR-205-5p	Upregulated	2.94	[167]
miR-200a	Upregulated Upregulated	N/A N/A	[144] [161]
miR-200b	Upregulated Upregulated Upregulated	N/A 5.2 N/A	[144] [160] [161]
miR-200c	Upregulated Upregulated Upregulated	N/A N/A 46.7	[144] [160] [163]
miR-1290	Upregulated	2.26	[169]
miR-141	Upregulated	N/A	[144]
miR-373	Upregulated	N/A	[161]
miR-30d	Downregulated	0.01–0.21 *	[165]
**Proteins**			
EGFR	Expressed	N/A	[149]
Serpin B5	Upregulated	N/A	[142]
Serpin C1	Downregulated	N/A	[153]
APOE	Upregulated Expressed	N/A N/A	[153] [149]
EpCAM	Upregulated Upregulated Upregulated	6.5 N/A N/A	[176] [153] [149]
FGA	Upregulated	N/A	[153]
FASN	Expressed	N/A	[149]
S100A14	Upregulated	N/A	[142]
S100A2	Upregulated	N/A	[142]
MYH11	Upregulated	N/A	[142]
CLCA4	Upregulated	N/A	[142]
IVL	Upregulated	N/A	[142]
CD109	Upregulated	N/A	[142]
NNMT	Upregulated	N/A	[142]
ENPP3	Upregulated	N/A	[142]
Plasminogen	Upregulated	N/A	[153]
C1q	Upregulated	N/A	[153]
PCNA	Expressed	N/A	[149]
TUBB3	Expressed Upregulated	N/A N/A	[149] [155]
CLDN3	Expressed	N/A	[149]
ERBB2	Expressed	N/A	[149]
L1CAM	Expressed	N/A	[149]
HSP70	Upregulated	3.43 ± 1.41	[152]
LBP	Upregulated	N/A	[153]
FGG	Upregulated	N/A	[153]
GSN	Downregulated	N/A	[153]
HGF	Upregulated	7–8	[154]
STAT3	Upregulated	4–5	[154]
IL6	Upregulated	2–3	[154]
AKT1	Upregulated	N/A	[155]
FAM49B	Upregulated	N/A	[155]
CLIC4	Upregulated	N/A	[155]
LTF	Upregulated	N/A	[155]
SNX3	Upregulated	N/A	[155]
URP2	Upregulated	N/A	[155]
MAP II	Upregulated	N/A	[155]
Claudin-4	Upregulated	N/A	[156]
TrkB	Upregulated	N/A	[157]
COL5A2	Upregulated	>2	[158]
LPL	Upregulated	>2	[158]
sE-cad	Upregulated	N/A	[159]

N/A: Not Available.

**Table 7:** EV-associated markers shared among different cancers.

miRNAs	Tumor	Up/Downregulation	References
miR-21	Ovarian cancer Prostate cancer Bladder cancer HPV-related cervical cancer Breast cancer	Upregulated Upregulated Upregulated Upregulated Upregulated Upregulated Upregulated	[46,62,86,88,118,123,124,160]
miR-21-5p	Urothelial carcinoma		
miR-145	Ovarian cancer Prostate cancer	Upregulated Upregulated	[130,163,167]
miR-126	Ovarian cancer Bladder cancer	Downregulated Ratio	[87,144]
miR-92a miR-92a-3p	Ovarian cancer Breast cancer Cervical cancer	Upregulated Upregulated Downregulated Upregulated	[47,65,160]
miR-92a-2-5p	Breast cancer		
Let7b Let7c Let7i	Ovarian cancer Prostate cancer Breast cancer	Downregulated Upregulated Downregulated	[33,123,165]
miR-320 miR-320a	Ovarian cancer Breast cancer	Downregulated Upregulated	[33,160]
miR-375	Bladder cancer Prostate cancer Ovarian cancer	Upregulated Upregulated Upregulated	[85,123,124,168]
miR-720	Ovarian cancer Bladder cancer	Downregulated Upregulated	[88,165]
miR-205 miR205-5p	Ovarian cancer Ovarian cancer Bladder cancer	Up or Downregulated Upregulated Upregulated	[88,144,165,167]
miR-200 miR-200 a/b/c miR-200c-3p	Prostate cancer Ovarian cancer Bladder cancer	Upregulated Upregulated Upregulated	[88,124,144,160,161,163]
miR-1290	Ovarian cancer Prostate cancer	Upregulated Upregulated	[126,168]
miR-141	Prostate cancer Ovarian cancer	Upregulated Upregulated	[127,133,144]
miR-373	Breast cancer Ovarian cancer	Upregulated Upregulated	[49,160]
miR-30-c-5p miR-30d	Renal cell carcinoma Ovarian cancer	Downregulated Downregulated	[70,165]
miR-1246	Breast cancer Prostate cancer	Upregulated Upregulated	[46,135]
miR-19 miR-19b-3p miR-19b1-5p	Prostate cancer Breast cancer Bladder cancer	Upregulated Upregulated Upregulated	[47,89,129]
miR-146a	Bladder cancer HPV-related cervical cancer	Upregulated Upregulated	[62,85]
miR-20a-5p miR-20b-5p	Cervical cancer Breast cancer	Downregulated Upregulated	[47,65]
miR-423 miR-423-3p	Breast cancer Cervical cancer	Downregulated Downregulated	[33,65]
LncRNAs			
MALAT1	Bladder cancer Cervical cancer	Upregulated Upregulated	[29,63]
HOTAIR	Cervical cancer Bladder cancer	Upregulated Upregulated	[63,90]
**Proteins**			
B-catenin D-catenin	Ovarian cancer Prostate cancer	Upregulated Expressed	[104,140]
EpCAM	Ovarian cancer Breast cancer	Upregulated Upregulated	[35,57,148,149,150]
FGA	Bladder cancer Ovarian cancer	Upregulated Upregulated	[77,153]
FASN	Prostate cancer Ovarian cancer	Expressed Expressed	[105,149]
LTF	Prostate cancer Ovarian cancer	Expressed Upregulated	[105,155]

## Data Availability

No new data were created or analyzed in this study. Data sharing is not applicable for this article.
